# Collaborative metabolic curation of an emerging model marine bacterium, *Alteromonas macleodii* ATCC 27126

**DOI:** 10.1371/journal.pone.0321141

**Published:** 2025-04-24

**Authors:** Daniel Sher, Emma E. George, Matthias Wietz, Scott Gifford, Luca Zoccarato, Osnat Weissberg, Coco Koedooder, Waseem Bashir Valiya Kalladi, Marcelo M. Barreto Filho, Raul Mireles, Stas Malavin, Michal Liddor Naim, Tal Idan, Vibhaw Shrivastava, Lynne Itelson, Dagan Sade, Alhan Abu Hamoud, Yara Soussan-Farhat, Noga Barak, Peter Karp, Lisa R. Moore

**Affiliations:** 1 Department of Marine Biology, Leon H. Charney School of Marine Sciences, University of Haifa, Israel; 2 Integrative Oceanography Division, Scripps Institution of Oceanography, University of California, San Diego, La Jolla, California, United States of America; 3 Alfred Wegener Institute Helmholtz Centre for Polar and Marine Research, Bremerhaven, Germany; 4 Max Planck Institute for Marine Microbiology, Bremen, Germany; 5 Department of Earth, Marine and Environmental Sciences, The University of North Carolina at Chapel Hill, Chapel Hill, North Carolina, United States of America; 6 Institute of Computational Biology, University of Natural Resources and Life Sciences, Vienna, Austria; 7 Core Facility Bioinformatics, University of Natural Resources and Life Sciences, Vienna, Austria; 8 The Fredy and Nadine Herrmann Institute of Earth Sciences, Hebrew University of Jerusalem, Jerusalem, Israel; 9 The Interuniversity Institute for Marine Sciences in Eilat, Eilat, Israel; 10 Israel Oceanographic and Limnological Research, Haifa, Israel; 11 University of Alabama at Birmingham, United States of America; 12 Department of Plant Pathology and Microbiology, Robert H. Smith Faculty of Agriculture, Food and Environment, The Hebrew University of Jerusalem, Rehovot, Israel,; 13 Zuckerberg Institute for Water Research, Ben-Gurion University of the Negev, Beer-Sheba, Israel; 14 Avram and Stella Goldstein-Goren Department of Biotechnology Engineering, Ben-Gurion University of the Negev, Beer-Sheva, Israel; 15 Department of Biomolecular Sciences, The Weizmann Institute of Science, Rehovot, Israel; 16 School of Zoology, Faculty of Life Sciences, Tel-Aviv University, Tel-Aviv, Israel; 17 Bioinformatics Research Group, SRI International, Menlo Park, California, United States of America.; Université de Perpignan: Universite de Perpignan Via Domitia, France

## Abstract

Inferring the metabolic capabilities of an organism from its genome is a challenging process, relying on computationally-derived or manually curated metabolic networks. Manual curation can correct mistakes in the draft network and add missing reactions based on the literature, but requires significant expertise and is often the bottleneck for high-quality metabolic reconstructions. Here, we present a synopsis of a community curation workshop for the model marine bacterium *Alteromonas macleodii* ATCC 27126 and its genome database in BioCyc, focusing on pathways for utilizing organic carbon and nitrogen sources. Due to the scarcity of biochemical information or gene knock-outs, the curation process relied primarily on published growth phenotypes and bioinformatic analyses, including comparisons with related *Alteromonas* strains. We report full pathways for the utilization of the algal polysaccharides alginate and pectin in contrast to inconclusive evidence for one-carbon metabolism and mixed acid fermentation, in accordance with the lack of growth on methanol and formate. Pathways for amino acid degradation are ubiquitous across *Alteromonas macleodii* strains, yet enzymes in the pathways for the degradation of threonine, tryptophan and tyrosine were not identified. Nucleotide degradation pathways are also partial in ATCC 27126. We postulate that demonstrated growth on nitrate as sole nitrogen source proceeds via a nitrate reductase pathway that is a hybrid of known pathways. Our evidence highlights the value of joint and interactive curation efforts, but also shows major knowledge gaps regarding *Alteromonas* metabolism. The manually-curated metabolic reconstruction is available as a “Tier-2” database on BioCyc.

## Introduction

Metabolism, the complex network of (mostly enzymatic) reactions within and between cells, underlies life on Earth. Reconstructing the metabolic network of an organism based on genomic information remains a fundamental challenge in biology [1,2] For model organisms like *Escherichia coli*, decades of physiological, biochemical, molecular and bioinformatic work have resulted in precise maps of cellular metabolism and its regulation [2]. These maps and accompanying biological knowledge form an integrated “encyclopedia of the cell”, such as the EcoCyc database, helping to explore cell metabolism and interpret experimental results [3]. Metabolic reconstructions (also termed Genome scale Network Reconstructions, or GENREs) also serve as basis for quantitative and mechanistic models of cell growth under different conditions (Genome scale Models, or GEM), such as those used in Flux Balance Analysis [2,4]. Curated metabolic databases are available for several medically and biotechnologically-relevant model bacteria such as *Salmonella enterica* [5] and *Bacillus subtilis* [6], as well as for selected eukaryotic organisms such as *Saccharomyces cerevisiae* (e.g., https://yeast.biocyc.org, [7]), *Arabidopsis thaliana* [8], and humans [9,10]. However, the vast majority of metabolic models for thousands of other organisms are derived purely from automatic pipelines for gene and pathway identification, without manual curation (e.g., [11–13]).

Although computational reconstructions are useful starting points for understanding cell metabolism, they are often incomplete or incorrect. For example, they may lack metabolic reactions encoded in the genome that were not identified by the computational pipelines that link genes to reactions and products. Furthermore, entire pathways can be incorrectly predicted (“false positives”) based on the presence of only some associated genes, especially if involved in multiple pathways [1]. Additionally, computational reconstructions lack the supervision of a human curator, who can consider supporting experimental evidence.

Manually curating a metabolic reconstruction, such as the “Tier-2” PGDBs (Pathway/Genome DataBase) available on BioCyc.org [13], comprises several stages. The initial metabolic reconstruction is computed from a published genome. In BioCyc, this is based on prior genome annotation using Pathway Tools (PTools) software [14] and MetaCyc [15] as the reference database for metabolic reactions. Gaps in the draft metabolic network are then filled by suggesting candidate genes (“pathway hole filling”, [16]). For BioCyc this stage includes the prediction of transport reactions [17] and operons, and imports protein features from UniProtKB [18] and Protein Database [19]. Finally, manual curation by one or more experienced curators includes correcting errors, updating gene and protein information, and summarizing the presence and function of enzymes, reactions and pathways based on the literature and experimental evidence. Supporting information includes gene knock-outs or naturally-occurring mutants, enzymatic activity assays, transcriptomics, and proteomics. The curation process also highlights needs for additional experimental verification of specific pathways. Because the manual curation process takes months to years, the BioCyc collection contains, as of Feb 2025, only 83 manually-curated (“Tier-1 and Tier-2”) PDGBs, compared to over 20,000 purely computationally generated ones (“Tier-3”). Thus, manual curation constitutes a significant bottleneck in consolidating knowledge on cellular metabolism, especially in emerging model organisms with environmental, biotechnological or medical potential, for which resources and data are limited.

One way of facilitating high-quality metabolic reconstructions is the joint effort by a community of researchers, either as a decentralized effort to which curators contribute remotely, or as an in-person curation workshop (e.g., “jamborees”, [20–22]). During the early days of genome sequencing, such community efforts were relatively common [21]. Today, such efforts focus on annotating individual genes in eukaryotes, and are facilitated by web portals such as Apollo, Jbrowse, ORCAE, and G-OnRamp [23–26]. Community annotation also serves to update the Gene Ontology database [27], and is an exciting way to involve undergraduates in bioinformatic research (e.g., [28,29]). Finally, community curation efforts with an emphasis on metabolic reconstruction have typically focused on medically or biotechnologically important model organisms such as yeast, trypanosomes or the bacterium *Salmonella typhimurium* [4,30,31]. Yet, these studies often lack concrete examples of the challenges encountered during manual curation, the decisions taken to resolve (as much as possible) these challenges, and the biological questions that emerge during this process.

Building upon the community curation approach [22], and extending it from individual genes to metabolic pathways, we organized an in-person community curation for the metabolic reconstruction of the marine bacterium *Alteromonas macleodii* ATCC 27126 (herein referred to as ATCC 27126). *Alteromonas macleodii* belongs to the ecologically and physiologically diverse genus *Alteromonas*, which is ubiquitous in tropical and temperate oceans and often abundant on particles [32–35]. *Alteromonas* are commonly associated with cyanobacteria [36–39] and algae [40,41]. *Alteromonas* strains are easily isolated and cultured, partly attributed to their rapid response to the availability of organic matter [42,43]. Indeed, a single *Alteromonas* strain has been shown to be capable of metabolizing almost the entire labile pool of marine organic carbon [44]. Phylogenetic, genomic and evolutionary studies have highlighted how genetic traits are exchanged between *Alteromonas* strains through genomic islands and plasmids (e.g., [45–50]). Some *Alteromonas* strains may also have biotechnological applications (e.g., [51,52]). Therefore, *A. macleodii* constitutes a relevant model organism in marine microbiology and biological oceanography [33]. The type strain, ATCC 27126, was isolated from surface seawater near Hawaii, and its physiology has been characterized in some detail [53,54].

**Fig 1:**
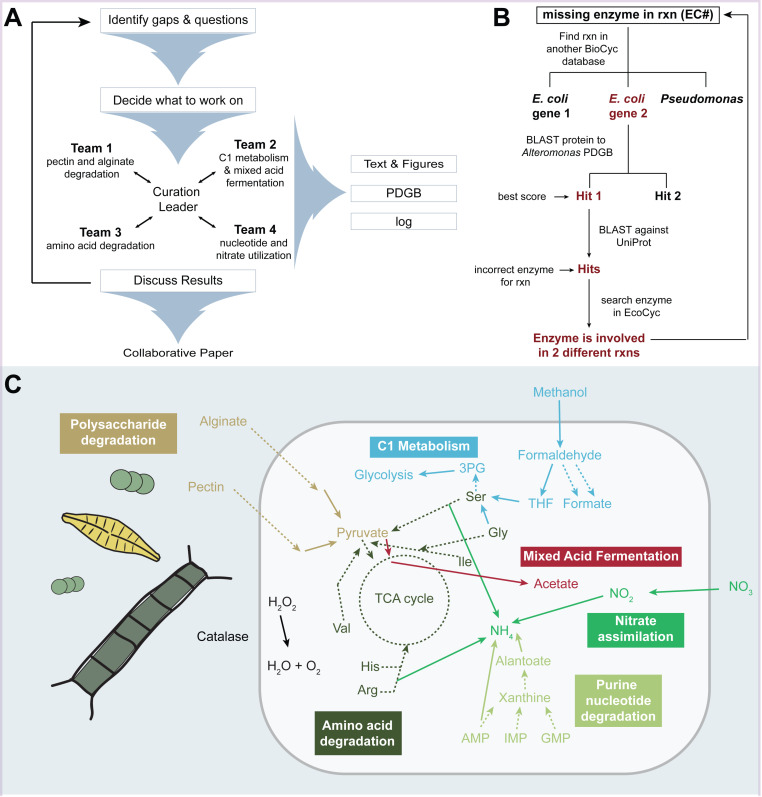
Schematic illustration of the **A)** **workshop structure,** **B)** **key curation methodology, and** **C)** **main pathways curated**.

Here, we describe conceptual and technical aspects of the community curation effort, performed at the University of Haifa (Israel) in February 2023 (Fig 1A). We specifically discuss the type of evidence typically available for emerging model organisms (Fig 1B), and then describe the curation of phenotypic traits with relevance for the ecological dynamics of *A. macleodii* (Fig 1C). We focused on pathways related to the uptake and utilization of carbon and nitrogen sources: 1) polysaccharides and one-carbon (C1) compounds, as well as mixed acid fermentation; 2) nitrate, nucleotides and amino acids. The metabolic reconstruction is available on BioCyc (PDGB ID: 2OKO). Furthermore, the 2023 version 27.5 is available freely on https://github.com/Sher-lab/amac/. This reconstruction serves as the basis for ongoing work developing a Genome scale Model (GEM) for *Alteromonas*.

### Metabolic curation − conceptual and technical aspects (Materials and Methods)

#### Automated metabolic reconstruction by PathwayTools.

The starting point for the collaborative, manual curation process was a “Tier-3” PGDB, computationally derived by PathwayTools [14]. Briefly, PathwayTools builds upon a genome annotated with the NCBI RefSeq PGAP pipeline [55] for consistency between PGDBs; but RAST [56], PROKKA [57] or DOE’s JGI/IMG [58] have also been used. The PathoLogic component of PathwayTools then infers the reactome of the organism, i.e., the set of enzyme-catalyzed reactions, by mapping genes to enzymatic reactions in the MetaCyc reference knowledgebase [15]. The linking is based on a combination of gene and/or gene product names, Enzyme Commission (EC) numbers and Gene Ontology (GO) terms, if included in the annotation [14]. The inferred reactome then allows inferring the presence of specific metabolic pathways, based on a likelihood score that considers the fraction of enzymes identified per pathway, the presence of pathway-specific enzymes, and the expected phylogenetic distribution (e.g., a plant-specific pathway suggested to be in a bacterium would be flagged and the score penalized). Only pathways above a defined threshold (likelihood score > 0.15) are inferred. As genes with unknown functions are common in model organisms (e.g., 35% in *E. coli*; [59]) and even more in non-model organisms (up to 80%; [60]), the pathway thresholding step is intentionally permissive. This allows pathways to be integrated into the predicted metabolic network even if enzymes are missing. Next, a pathway hole filler (PHFiller) within PathoLogic identified reactions with no associated enzyme, and attempts to fill these holes (identify the needed enzyme) using a BLAST search with multiple candidate genes from UniProtKB [16]. The resulting PDGB includes a report showing the score and completeness for each pathway, pathway holes that were “filled”, and pathways with remaining holes. This enables assessing the quality of the metabolic reconstruction by the curators, and advises where to perform manual curation − representing a robust quality control of the predicted metabolic network, where available experimental evidence is added via comments and evidence codes.

#### Community curation.

Manual curation of the ATCC 27126 metabolic network was mostly performed during a four-day workshop by diverse researchers, including graduate students, postdocs and PIs under the guidance of a BioCyc curator (Lisa R. Moore) (Fig 1A). All workshop participants are co-authors on this paper. Prior to the curation workshop, a five-day course introduced the fundamentals of metabolic reconstruction and downstream uses, e.g., interpreting ‘omics data in light of metabolism. The workshop participants decided on the priorities for curation, taking into account the research interest in *Alteromonas* as versatile utilizers of dissolved and particulate organic matter, and their interactions with phytoplankton. Subteams of 3–5 curators focused on the curation of one or more pathways using the curation interface of the BioCyc web tool (unpublished). Often, multiple pathways were combined for visual interpretation using the “Pathway Collage” tool in BioCyc [61].

#### Evidence types.

Most organisms whose PDGBs undergo manual curation are widely studied; often being genetically tractable and/or medically important taxa. Such organisms usually have accompanying gene-specific information, such as knock-out phenotypes or biochemical assays with purified proteins. In contrast, emerging model organisms often lack such information, and may not be genetically tractable (e.g., *Prochlorococcus* strains MED4 and SS120 with Tier-2 PDGBs available in BioCyc). ATCC 27126 was initially described in 1972 [53], yet knock-out phenotypes have been only described for genes encoding a nitrate reductase and siderophore synthesis proteins [62,63]. As a result, we considered additional types of evidence for metabolic reconstruction. Firstly, we compiled a list of media on which ATCC 27126 can grow, based on published studies as well as experiments performed for the curation workshop (S1 File). Since ATCC 27126 can grow on minimal media with C, N, P and Fe sources but without amino acids, vitamins or cofactors; complete pathways for producing these compounds must be present. Such information was added as metadata to the specific pathway descriptions in the PGDB. Secondly, we considered evidence from related *Alteromonas* strains. For example, polysaccharide utilization pathways were curated through comparison with *A. macleodii* 83–1, a model polysaccharide degrader with 98% average nucleotide identity to ATCC 27126 [64]. Finally, we identified candidate genes filling a specific pathway hole using Reciprocal Best BLAST (RBBH, [65], Fig 1B), using candidate “hole filling” genes identified in MetaCyc or EcoCyc using the EC number for each missing reaction. The protein sequence (from *E. coli* or, if using MetaCyc, from the closest relative of *Alteromonas*) was then queried against the PGDB using BLASTP within BioCyc. The best hit in ATCC 27126 was queried against the UniProtKB/Swiss-Prot database [18]. A gene product in ATCC 27126 was considered as RBBH (i.e., fill a pathway hole) if the best hit in UniProtKB/Swiss-Prot was annotated as the same function or EC number as the initial MetaCyc/EcoCyc query. In some cases, additional information was considered, such as the specificity of the annotation (e.g., methanol dehydrogenase vs. dehydrogenase), or BLAST sequence similarity and query cover. For Fig 3C, multiple sequence alignments were performed using MAFFT [66]. Maximum likelihood trees of *Alteromonas* alcohol dehydrogenases and 16S rRNA genes, aligned with MUSCLE in AliView, were inferred using IQ-TREE v1.5.4 [67–69].

**Fig 2:**
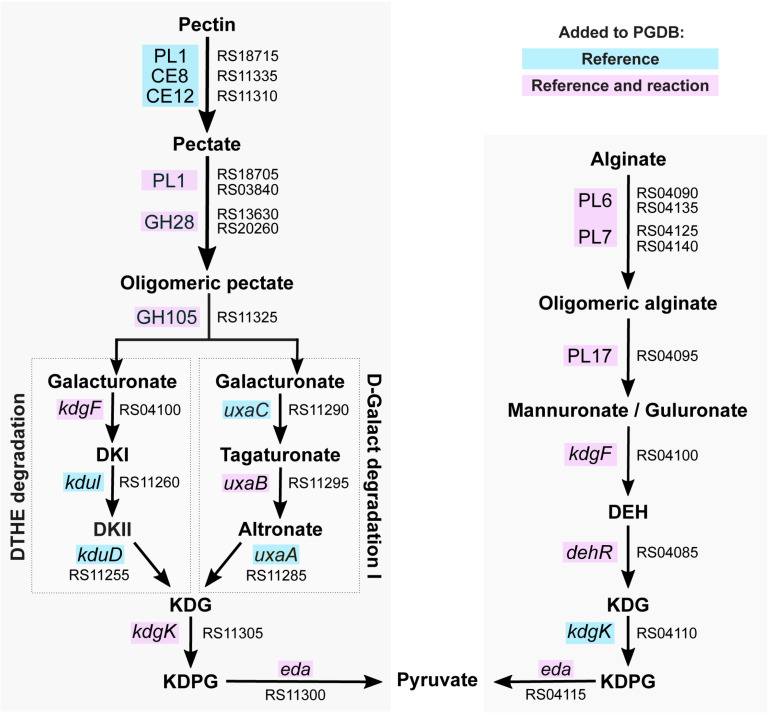
Curated pectin and alginate degradation pathways. The pectin superpathway (left; PWY2OKO-5) encompasses initial depolymerization and demethylation followed by 4-deoxy-L-threo-hex-4-enopyranuronate degradation (PWY-6507; abbreviated DTHE) or D-galacturonate degradation I (GALACTUROCAT-PWY; abbreviated D-Galact) for unsaturated and saturated galacturonates respectively. Both pectin and alginate degradation (right; PWY-6986-1) eventually result in KDG, KDGP and pyruvate, but these metabolites are generated via dedicated enzymes. DKI: 5-keto-4-deoxyuronate; DKII: 2,5-diketo-3-deoxygluconate; DEH: 4-deoxy-l-erythro-5-hexoseulose uronate; KDG: 2-keto-3-deoxygluconate; KDPG: 2-dehydro-3-deoxy-D-gluconate 6-phosphate. Blue boxes indicate genes for which the reference information was updated in the PDGB. Pink boxes indicate genes for which new reactions were added. Gene names and locus tags are shown for each reaction.

## Results and discussion

The metabolic curation of ATCC 27126 was performed using a Tier-3 PDGB generated from NCBI genome assembly GCF_000172635.2. The reactions and pathways were then curated as described above, resulting in a Tier-2 PDGB (Table 1). The supplementary Excel File provides a detailed log of all steps within the collaborative curation, as well as a comparison between the initial reconstructions performed through BioCyc, Carveme and Kbase [12,70].

**Table 1:** Summary statistics of the curated Tier-2 PGDB of *Alteromonas macleodii* ATCC 27126, version 27.5.

Genes:	3,962
Pathways:	244
Enzymatic Reactions:	1,492
Transport Reactions:	19
Polypeptides:	3,829
Protein Complexes:	49
Enzymes:	915
Transporters:	304
Compounds:	1,029
Transcription Units:	2,659
tRNAs:	52
Protein Features:	6,181
GO Terms:	4

The manual curation corrected several errors produced by the automatic annotation, and raised some interesting biological questions (Fig 1C): 1) The automatic reconstruction predicted that ATCC 27126 could perform mixed acid fermentation, but our results cast doubt on this inference; 2) ATCC 27126 was predicted to utilize all amino acids as sole carbon sources, yet we identified several pathway holes; 3) ATCC 27126 was predicted by the automatic curation not to utilize nucleotides, yet we show that much of the pathway for nucleotide degradation is present, and predict the release of specific metabolites; 4) ATCC27126 was predicted to perform denitrification, yet we show that this is likely incorrect, and that this strain likely performs assimilatory nitrate reduction. Below we discuss the main pathways and processes curated in more detail.

## Carbon sources

### Carbohydrate-active enzymes and polysaccharide degradation

ATCC 27126 encodes several pathways to degrade algal polysaccharides, important bacterial nutrient sources in the oceans. Degradation relies on polysaccharide lyases (PL), glycoside hydrolases (GH) and carbohydrate esterases (CE). These genes are often encoded in polysaccharide utilization loci (PULs), operon-like gene clusters with concerted regulation. Here, using complementary evidence, we curated the pathways for pectin and alginate degradation (Fig 2). We annotated genes encoding carbohydrate-active enzymes (CAZymes, [71,72]) in ATCC 27126 in light of transcriptomic and proteomic data from a closely related strain, *A. macleodii* 83–1. Both strains harbor homologous alginolytic and pectinolytic PULs [64,73], which are significantly upregulated in 83–1 when growing with an alginate and pectin mix [64]).

For pectin degradation, we imported relevant degradation subpathways from MetaCyc and constructed a new pectin superpathway, PWY2OKO-5, encompassing depolymerization (via PL1, GH28 and GH105) and demethylation (via CE8 and CE12) (Fig 2). The resulting galacturonates are then processed via 4-deoxy-L-threo-hex-4-enopyranuronate (PWY-6507) and D-galacturonate (GALACTUROCAT-PWY) pathways respectively, which we added to the ATCC 27126 PDGB. The released methanol is possibly metabolized by alcohol dehydrogenases (see below).

Curating the alginate degradation pathway benefited from RT-qPCR evidence in ATCC 27126, showing significantly higher expression of PL6 and PL7 lyases with alginate as sole nutrient source [73]. Biochemical assays in 83–1 with cloned, homologous enzymes confirmed alginate lyase activity, and characterized salinity and temperature optima [74]. However, structural elucidation failed, since not enough soluble enzyme was obtained [74]. Our curation process involved adding reactions for 4-deoxy-l-erythro-5-hexoseulose uronate (DEH) reductase (alginate degradation) as well as *kdgF* (both pathways), since uronate conversion does not occur spontaneously [75] as originally annotated in BioCyc (Fig 2).

Both pectin and alginate are composed of uronate sugars, eventually yielding pyruvate from 2-keto-3-deoxygluconate (KDG) and 2-dehydro-3-deoxy-D-gluconate 6-phosphate (KDPG, Fig 2). Notably, KDG and KDPG are generated via pectin- or alginate-specific *kdgK* and *eda* genes encoded in the respective PULs for each polysaccharide. ATCC 27126 encodes another *eda* copy (MASE_RS11155) not induced by pectin or alginate in 83–1, which might be a “generic” variant to convert KDPG from other sources.

### C1 metabolism

**Fig 3:**
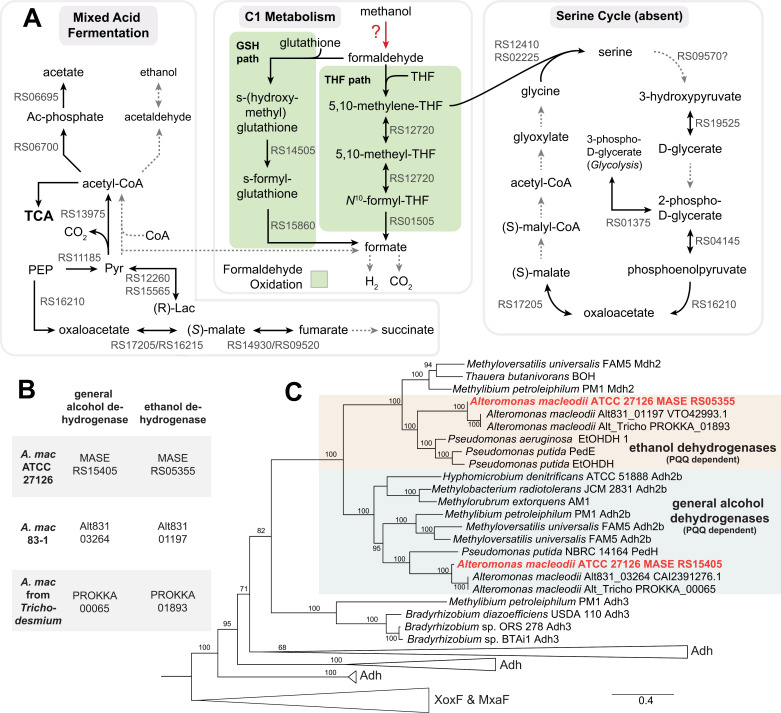
C1 metabolism, the serine cycle and mixed acid fermentation in *A. macleodii* ATCC 27126. A) Potential routes for one-carbon and methylated compounds. Bold arrows represent reactions identified in the ATCC 27126 genome, dotted arrows are missing reactions. THF: tetrahydrofolate; Lac: lactate; Pyr: pyruvate; PEP: phosphoenolpyruvate; GSH- reduced glutathione. B) Putative methanol/ethanol dehydrogenase genes in *Alteromonas* strains that grow and do not grow on methanol. C) Maximum likelihood tree (IQ-TREE) inferred under the Q.pfam+F+I+G4 model from alcohol dehydrogenase proteins from ATCC 27126 and other strains. Support values represent 100 bootstrap pseudoreplicates.

One-carbon (C1) and methylated compounds are important bacterial substrates in the marine environment [76,77]. Here we examined the potential of ATCC 27126 to metabolize methanol, formaldehyde (the central C1 intermediate), formate, and related cofactors and enzymes (Fig 3A).

#### Methanol/Ethanol Dehydrogenases.

Methanol is commonly produced by phytoplankton and cyanobacteria [78], but only some *A. macleodii* strains can grow on methanol. Several *A. macleodii* strains were isolated from *Trichodesmium* using media with methanol as the sole carbon source, attributed to pyrroloquinoline quionone (PQQ)-dependent alcohol dehydrogenases (ADHs) encoded in their genomes [79]. In contrast, neither ATCC 27126 nor 83–1 grow on methanol ([54,64]), and our growth results), although both encode the same PQQ-dependent ADH genes (Fig 3B). ATCC 27126 and 83–1 also harbor an operon (*pqqABCDE*) encoding the PQQ cofactor; this metabolite was identified using mass spectrometry in 83–1 [64]. Our phylogenetic analysis of predicted PQQ-dependent ADH genes did not support their potential role in methanol oxidation (Fig 3C): MASE_RS15405 is within a clade of general ADHs that could potentially mediate methanol oxidation, while MASE_RS05355 is more related to ethanol dehydrogenases. Additionally, both genes show only moderate amino acid identity (30%) with the known PQQ-dependent methanol dehydrogenases XoxF and MxaF [80]. These ADHs might alternatively convert ethanol to acetaldehyde; indeed, ATCC 27176 was shown to grow on ethanol [54].

ATCC 27126 also encodes a zinc-dependent ADH (MASE_RS02430) and three iron-containing ADHs (MASE_RS06555, MASE_RS01350, MASE_RS11390). Iron- and zinc-containing ADHs can catalyze the oxidation of methanol, ethanol, and other alcohols (e.g., [81–83]), although some of these enzymes may preferentially catalyze the reverse reaction (i.e., reduction; see mixed acid fermentation below). Alternatively, both ethanol and methanol can be converted to acetaldehyde and formaldehyde, respectively, during hydrogen peroxide detoxification by catalase and related enzymes (e.g., [84]). ATCC 27126 encodes five catalase genes that potentially mediate interactions with phytoplankton [85], yet it is unclear whether this pathway (primarily for detoxification) produces significant energy to support growth.

In summary, despite finding several candidate genes for methanol oxidation, it remains unclear why ATCC 27126 does not grow on methanol while other strains are able to do so. Potentially, ATCC 27126 can metabolize methanol, but not grow on it as a sole C source. The same has been shown for *Pelagibacter,* which encodes multiple genes involved in C1 metabolism (including ADHs), oxidizes methanol and other C1 compounds to CO_2_, yet does not incorporate C1 compounds into biomass [86]. Therefore, varying abilities between *A. macleodii* strains in their ability to utilize methanol may be due to different downstream oxidation steps.

#### Formaldehyde Metabolism.

Formaldehyde is a common byproduct of methanol oxidation and a critical intermediate in C1 metabolism. It is also often cytotoxic and must be metabolized quickly. ATCC 27126 is predicted to encode two parallel routes for formaldehyde oxidation: the tetrahydrofolate-based (THF) and the glutathione-based (GSH) pathway (Fig 3A).

The THF pathway comprises four steps leading to formate; the first step is spontaneous [87,88] and the remaining three catalyzed [89]. The product of the first step, 5,10-methylenetetrahydrofolate, can either enter the serine cycle (as in the methylotroph *Methylorubrum extorquens*, [90]) or be converted into formyltetrahydrofolate (as in the facultative methylotroph *Bacillus methanolicus,* which lacks the serine cycle [89]). As discussed below, ATCC 27126 likely does not encode the serine cycle, suggesting the presence of dissimilatory formaldehyde conversion via the THF pathway, yielding formate.

The GSH pathway involves NAD, glutathione-dependent formaldehyde dehydrogenase (GSH-FDH, also called S-(hydroxymethyl)glutathione dehydrogenase), and S-formylglutathione hydrolase (FGH). S-(hydroxymethyl)glutathione, formed spontaneously by formaldehyde and glutathione, is the preferred *in vitro* and presumed *in vivo* substrate for GSH-FDH [91,92]. Both GSH-FDH and FGH are encoded in the ATCC 27126 genome (MASE_RS14505 and MASE_RS15860, respectively). ATCC 27126 may need both THF and GSH pathways for C1 metabolism and/or detoxification of formaldehyde, as in *Methylorubrum extorquens* [90].

#### Formate metabolism.

Formate from formaldehyde oxidation is usually oxidized to CO_2_ [93], catalyzed by a formate hydrogen lyase complex (FHL) comprised of formate dehydrogenase *fdhF* and six subunits of hydrogenase 3. A BLAST search of *E. coli fdhF* (UniProt:P07658) showed several homologs in marine bacteria (mainly *Shewanella*), but no clear homolog in *Alteromonas*. An alternative route is the formate dehydrogenase operon (FDH), but we found only one of the four FDH genes (*fdhD*) in the ATCC 27126 genome. Although *fdhD* is encoded adjacent to another gene (MASE_RS07820) distantly related to a formate dehydrogenase (~31% identical to *fdhH* from *E. coli*), MASE_RS07820 is a pseudogene due to a frame shift. Therefore, ATCC 27126 presumably cannot oxidize formate to CO_2_.

Formate also participates in the glutamylation of tetrahydrofolate. Glutamylated folate cofactors are required in various C1 reactions, acting as carriers of one-carbon units [94]. However, the first enzyme in this reaction, formate-tetrahydrofolate ligase, is not encoded in the ATCC 27126 genome. The lack of key metabolic pathways supports the observation that *A. macleodii* cannot grow with formate as a sole carbon source ([53], and our growth results). Future work could determine whether ATCC 27126 excretes formate, similar to some methylotrophs [95] and cyanobacteria [96–98], potentially providing a carbon source for co-occurring organisms.

#### Serine cycle.

Formaldehyde can also be assimilated via the serine cycle (formaldehyde assimilation I pathway in BioCyc), yielding several intermediates for central carbon metabolism ([99], Fig 3A). In the first step, hydroxymethyltransferase (GlyA) catalyzes the reaction of 5,10-methylenetetrahydrofolate with glycine to form serine. ATCC 27126 encodes two GlyA proteins along with a putative serine-glyoxylate transaminase, an enzyme that mediates the following conversion of serine to 3-hydroxypyruvate. Hydroxypyruvate reductase, reducing 3-hydroxypyruvate to D-glycerate, was not identified in ATCC 27126. Instead, we found 2-hydroxyacid dehydrogenase, which also converts 3-hydroxypyruvate to D-glycerate. Genes encoding the remaining essential enzymes were not detected, including EC 6.2.1.9 and EC 4.1.3.24 that define the presence of the serine cycle in methylotrophs. This genomic evidence, along with the lack of growth on methanol as a sole carbon source ([64] and our results) supports the absence of the serine cycle in ATCC 27126.

### Mixed acid fermentation

Mixed acid fermentation involves the catabolism of pyruvate to lactate, formate, acetate, ethanol and succinate when no exogenous electron acceptors are available. Marine particles, to which *Alteromonas* is often attached (e.g., [32,100], potentially have microaerobic or anaerobic niches [101], yet. ATCC 27126 has been described as strictly aerobic [53]. Mixed acid fermentation was inferred in ATCC 27126 during the computational reconstruction of PDGB, albeit with pathway holes. Hence, we decided to investigate this pathway further.

ATCC 27126 encodes 7 out of 11 enzymes for mixed acid fermentation, including those catalyzing the conversions of acetyl-CoA to acetyl phosphate and acetate, pyruvate to lactate, as well as the formation of fumarate from phosphoenolpyruvate (Fig 3A). The presence of these reactions is supported by detecting acetate, lactate and succinate in extracellular polysaccharides (EPS), where they act as non-carbohydrate substituents [52,102]. However, ATCC 27126 does not encode a fumarate reductase enzyme. Thus, although traces of succinyl have been reported in *Alteromonas* EPS [52] and fumarate reduction can principally occur via succinate dehydrogenase [103], presence of mixed-acid fermentation in ATCC 27126 remains unclear. Moreover, ATCC 27126 lacks the genes catalyzing the initial anaerobic conversion of pyruvate and CoA to acetyl-CoA and formate (*pflB* and *tdcE* in *E. coli* K-12). As discussed above, it also lacks the formate dehydrogenase complex that catalyzes the sequential conversion of formate to CO_2_. Finally, ATCC 27126 lacks the canonical genes catalyzing the reduction of acetyl-CoA to acetaldehyde and ethanol. While, in principle, acetaldehyde could be reduced to ethanol by one of the PQQ- or zinc-dependent alcohol dehydrogenases (described above) working in reverse, the lack of an acetaldehyde dehydrogenase gene suggests that this part of mixed acid fermentation may be dysfunctional. Therefore, the bioinformatic evidence for the full mixed acid fermentation process is inconclusive. Nonetheless, many of these reactions also work in reverse (e.g., lactate dehydrogenase), and may enable ATCC 27126 to catabolize organic acids excreted by co-occurring algae even under oxic conditions [98,104]. Indeed, ATCC 27126 can grow on lactate or pyruvate as sole carbon sources ([54], S1 File).

## Nitrogen sources

### Amino acid degradation

**Fig 4:**
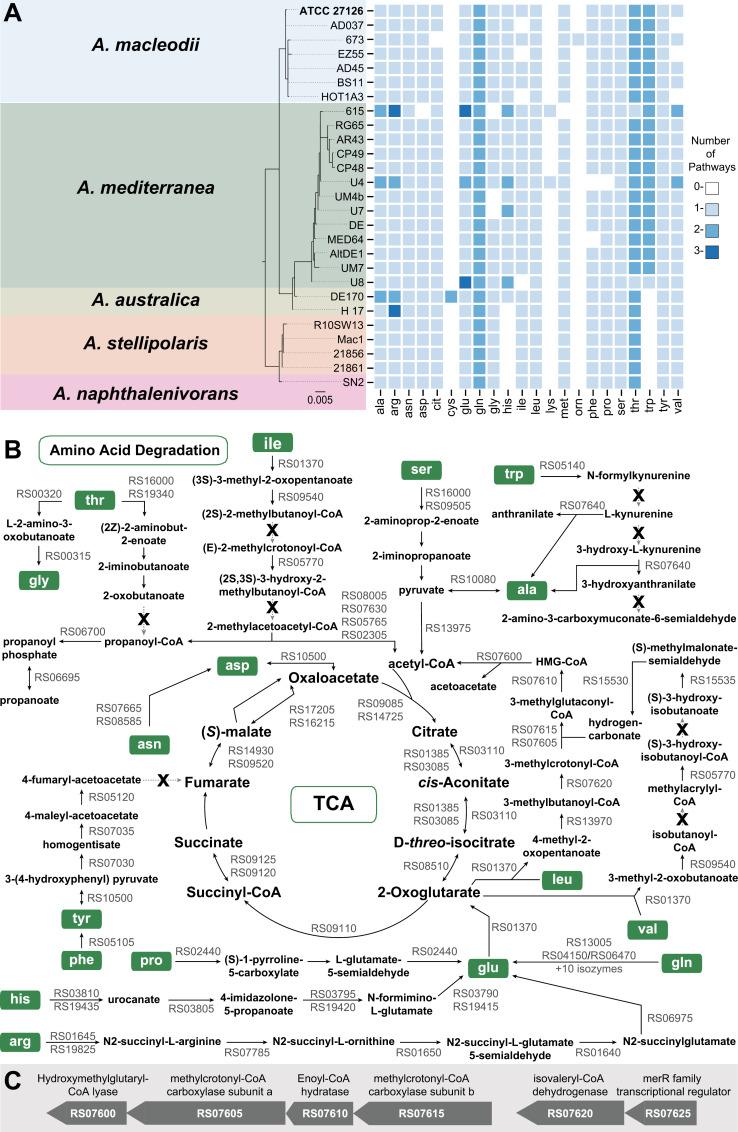
Amino acid degradation pathways and their holes. A) Number of predicted amino acid degradation pathways per genome across *Alteromonas* spp., determined using BioCyc’s Comparative Genomics tool. The taxa are ordered based on a maximum likelihood tree (IQ-TREE) inferred under the TIM3+F+I model from full-length 16S rDNA. B) Amino acid utilization pathways in ATCC 27126, drawn based on an overview from the BioCyc “Pathway Collage” tool. Missing reactions (“pathway holes”) are highlighted by an X. The reaction marked with a double line (RS15535, Valine degradation) has unknown directionality according to BioCyc. Methionine degradation is not shown as it enters the TCA cycle via multiple other pathways. C) Predicted operon for branched chain amino acid degradation. RS07605 and RS07615 encode two subunits of the methylcrotonyl -CoA carboxylase, with a predicted “hole-filling” hydratase between them, preceded by RS07620 (isovaleryl-CoA dehydrogenase) and followed by RS07600 (Hydroxymethylglutaryl-CoA lyase). The operon organization is similar to the *liu* operon in *Pseudomonas aeruginosa* [107].

Amino acids, constituting a significant fraction of organic nitrogen in the oceans [105], can serve as both nitrogen and carbon sources. ATCC 27126 grows well with peptides or a mixture of amino acids as sole carbon sources [106]. Although comparative genomics suggested that most *Alteromonas* spp. can degrade almost every amino acid (Fig 4A), ATCC 27126 growth was only reported on alanine and glycine [54]. We asked whether this inconsistency is due to missing genes in the predicted amino acid degradation pathways as they enter the TCA cycle (Fig 4B). For seven pathways with putative holes, we identified candidate genes for three pathways that could “close” these holes using RBBH (S1 File). The putative “hole-filling” genes MASE_RS07620 and MASE_RS07610 are encoded within a predicted operon for L-leucine degradation, somewhat similar to the *liu* operon from *Pseudomonas aeruginosa* (Fig 4C, [107]). The predicted “hole-filling” gene MASE_RS01650 is part of an operon for arginine degradation. However, we could not reconstruct the pathways for the degradation of threonine, tryptophan and tyrosine. Notably, there are holes in the pathways for tryptophan and tyrosine degradation in *E. coli* SIJ488, yet this strain can utilize these amino acids as sole nitrogen sources [108]. Therefore, these pathways might still be functional in ATCC 27126, although further experimental work is needed to test this hypothesis.

### Nucleotide degradation

**Fig 5:**
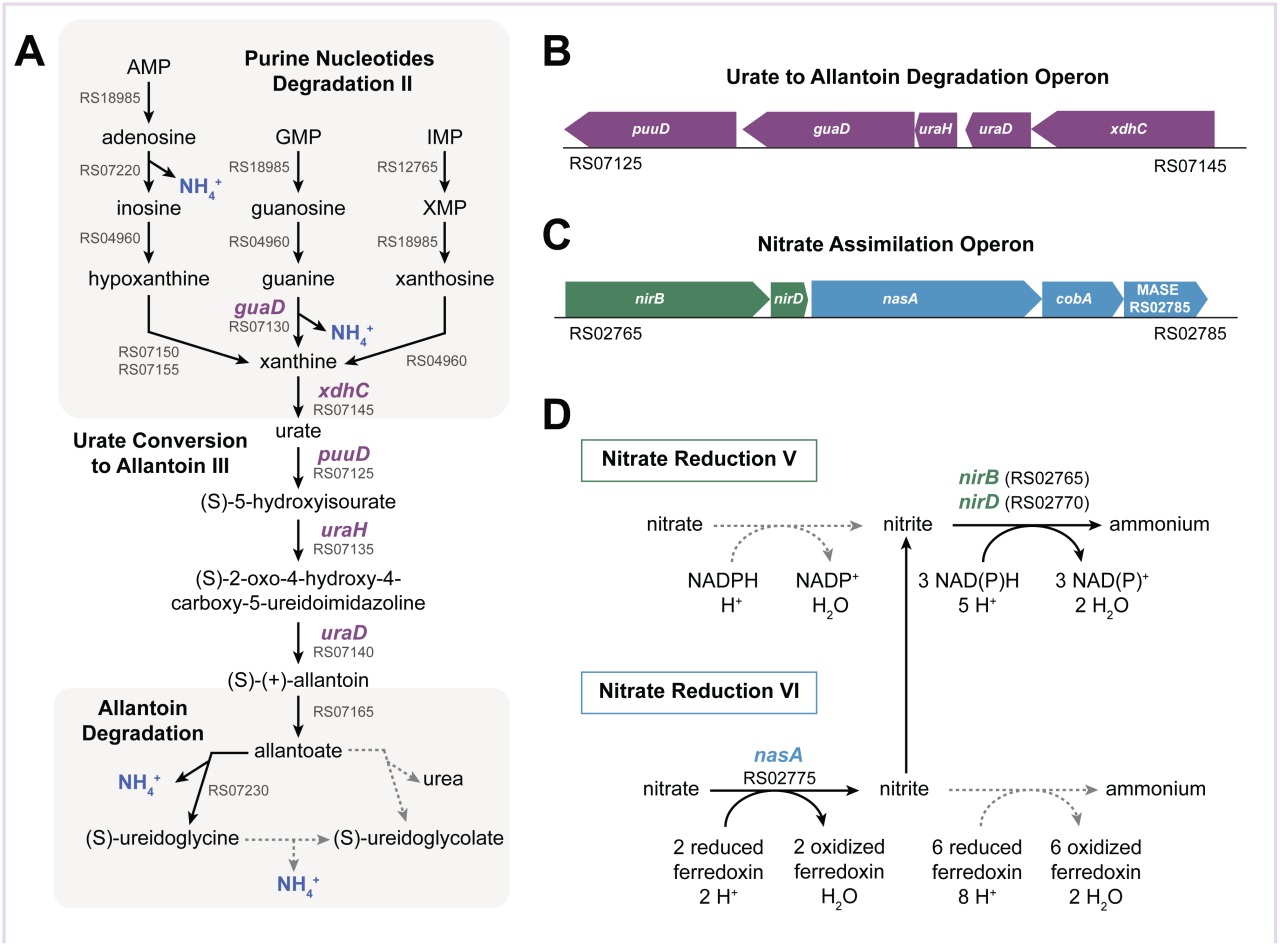
Nucleotide and nitrate utilization pathways. A) The purine nucleotide degradation **II** (aerobic) superpathway in ATCC 27126. Dotted arrows show missing reactions; stages where NH_4_ is released are highlighted. B) Genomic region surrounding the putative urate to allantoin degradation operon (*puuD* homologue). C) Putative assimilatory nitrate reductase operon. D) Suggested hybrid nitrate reductase pathway in ATCC 27126, utilizing different cofactors for nitrate and nitrite reduction.

Purine nucleotides can serve as nitrogen sources for bacteria [109]. The purine nucleotide degradation II superpathway in MetaCyc is composed of three sequential pathways: 1) purine nucleotide degradation II (starting with AMP, GMP and IMP each yielding urate); 2) urate conversion to allantoin; and 3) allantoin degradation (Fig 5A). The first pathway is completely encoded by ATCC 27126 (Fig 5A), whereas the second was not predicted despite finding genes encoding 2 out of 3 reactions. However, RBBH using the *puuD* gene from *Agrobacterium fabrum* identified MASE_RS07125 as the putative hole-filling gene in the urate conversion to allantoin pathway, encoding a protein containing a urate oxidase domain (PF016181) (Fig 5B). We therefore curated the *puuD* gene, and added the missing urate conversion pathway.

Further metabolism of allantoin to glyoxylate can occur via S-ureidoglycine or S-ureidoglycolate (Fig 5A). However, these pathways are incomplete in ATCC 27126, and we were unable to identify hole-filling genes. Accordingly, ATCC 27126 cannot grow on allantoin as sole C source [54]). Taken together, the missing allantoin degradation pathway might explain why ATCC 27126 cannot grow on nucleotides as sole carbon source [54]. Nevertheless, this strain might use purines as N sources, since ammonium is released at multiple steps of the degradation pathway. If so, we predict ATCC 27126 to excrete allantoin, allantoate and/or S-ureidoglycolate during nucleotide degradation.

### Nitrate assimilation

ATCC 27126 can grow on nitrate as a sole N source, likely incorporated to biomass via assimilatory reduction [62]. The draft PDBG did not include assimilatory, but rather dissimilatory, nitrate reduction (i.e., denitrification), via a cluster of one nitrate reductase (*nasA*; MASE_RS02775) and two nitrite reductase genes (*nirBD*, MASE_RS02765 and MASE_RS02770; Fig 5C). A *nasA* mutant of ATCC 27126 cannot grow on nitrate, highlighting that this operon encodes assimilatory nitrate reduction [62]. Additionally, there is no evidence for denitrification [54], although distantly related Alteromonads may respire nitrate [110]. Therefore, dissimilatory nitrate reduction was removed from the PDGB.

The putative assimilatory nitrate reduction pathway in ATCC 27126 shows an unusual combination of genes and required cofactors (Fig 5D). In most bacteria and fungi, nitrate and nitrite reductases use NAD(P)H as electron donor [111], whereas cyanobacteria use ferredoxin [112]. The ATCC 27126 *nasA* is most similar to homologs of the nitrate reduction V pathway common to many bacteria and fungi (BLAST bit score 678). However no homolog was found for the *nasC* protein, often associated with *nasA* to form the nitrate reduction complex [111]. The second best hit for *nasA* was to a cyanobacterial nitrate reductase *narB* (EC 1.7.7.2, BLAST bit score 607 with *narB* from *Synechococcus elogantus*), which does not require additional subunits, has a similar length, and shares ferredoxin-binding and molybdopterin-containing Pfam domains with ATCC 27126 *nasA*. We therefore propose that ATCC 27126 encodes a “chimeric” nitrate assimilation pathway, with a ferredoxin-utilizing homolog of the cyanobacterial nitrate reductase *narB* followed by NAD(P)H-utilizing bacterial homologs of the nitrite reductase *nirBD* complex. This hypothesis requires experimental verification.

## Conclusions and future prospects

*Alteromonas* are highly versatile degraders of organic carbon in the oceans [44], yet the factors that determine their “metabolic niche” and the drivers of their spatial and temporal dynamics remain unclear [32,35]. Our interactive community curation explored specific traits related to *Alteromonas* metabolism and ecology, providing recommendations for future experimental work, and consolidating the biological understanding of *A. macleodii* − hopefully resulting in a dynamic and growing “metabolic encyclopedia”. Specifically, we show that ATCC 27126 and other *A. macleodii* strains can utilize complex polysaccharides, but cannot grow on methanol or formate. Polysaccharide degradation might support the association of *Alteromonas* strains with algae and polymer microgels [113]. Similarly, growth on peptides [106], together with their ability to utilize various forms of dissolved organic phosphorus [114], may support their growth on other forms of organic matter such as dead jellyfish biomass [43] or deep-sea particles [115]. In contrast, ATCC 27126 cannot grow on chitin [54], the most abundant polysaccharide in zooplankton; indeed, *Alteromonas* are not part of the core copepod microbiome [116]. Future curation efforts and accompanying experiments focusing on carbohydrate, protein and organophosphorus degradation are needed to test these hypotheses.

The finding of *Alteromonas* on marine particles [32–34] might be connected to hypoxic or anoxic niches [101], yet the evidence for fermentation in ATCC 27126 is inconclusive. We propose that a better characterization of the “oxygen niche” of *Alteromonas* will help to understand their involvement in particle colonization and degradation [115]. Similarly, understanding how *Alteromonas* utilizes amino acids, nucleotides or nitrate will contribute to clarifying their role in nitrogen cycling, given that proteins can comprise 50–60% of phyto- and zooplankton biomass [105,117–120]. Importantly, comparing cultured strains with environmental datasets will need to consider the diversity among *Alteromonas* strains (e.g., [48–50,121].

Our community curation clarified important aspects of the physiology and ecology of ATCC 27126, and suggested relevant experimental directions. However, we also highlight key challenges in studying ecologically important but less described organisms. Only a few *A. macleodii* genes have been functionally verified, resulting in often indirect evidence for the presence or absence of specific reactions. Deciding whether or not to include pathways in the PDGB was therefore sometimes subjective, especially if requiring “hole filling”. Furthermore, manual curation requires in-depth knowledge of multiple aspects of metabolism, which is typically beyond the expertise of any single curator or a diverse group like in our study. We suggest that any curation process clearly records the evidence used to decide whether a pathway is present, enabling future users to revisit the metabolic reconstruction before generating genome-scale models. Secondly, there are no clear guidelines for the incorporation of genomic information for metabolic reconstructions. While the conservation of (partial) pathways in closely related organisms may suggest they are functional, our results for amino acid utilization highlight that such hypotheses are not always fully supported. Furthermore, systematically addressing the correlation between pathway phylogeny and function may facilitate metabolic curation by harvesting experimental results from yet-uncultured organisms, e.g., using metagenome-assembled or single-cell genomes [43]. Finally, a metabolic reconstruction widely accessible to the scientific community needs to be compatible with downstream analyses. There are currently several frameworks for representing cell metabolism, e.g., KEGG [122] and modelSEED [123], yet translating metabolic reconstructions from between frameworks can be difficult since terms and structure (e.g., where do they draw boundaries between pathways) are not compatible. Moreover, due to the cost of maintaining the BioCyc infrastructure, accessing most PDGBs requires payment. We hope that the insights from our community curation will provide incentives for research groups and funding agencies to include metabolic knowledge for environmentally important organisms in financially supported databases.

## Supporting information

S1 File Supplementary excel file - information on metabolic reconstruction. (XLSX)
